# Efficacy of Arthroscopic Shavers for the Retrieval and Processing of Connective Tissue Progenitor Cells from Subacromial Bursal Tissue

**DOI:** 10.3390/jcm11051272

**Published:** 2022-02-25

**Authors:** Ian J. Wellington, Benjamin C. Hawthorne, James C. Messina, Matthew R. LeVasseur, Mary Beth McCarthy, Mark P. Cote, Augustus D. Mazzocca

**Affiliations:** Department of Orthopedics, University of Connecticut, Farmington, CT 06032, USA; bhawthorne@uchc.edu (B.C.H.); jmessina@uchc.edu (J.C.M.); mlevasseur@uchc.edu (M.R.L.); mccarthy@uchc.edu (M.B.M.); mcote@uchc.edu (M.P.C.); mazzocca@uchc.edu (A.D.M.)

**Keywords:** bursa, arthroscopy, shaver, biologic, augmentation

## Abstract

The purpose of this study is to determine if arthroscopic shavers can effectively collect and process connective tissue progenitor (CTP) cells from subacromial bursal tissue for utilization in rotator cuff repair augmentation. Subacromial bursal tissue was collected and processed using two arthroscopic shavers, Shaver A and Shaver B, in 10 patients undergoing arthroscopic rotator cuff repair. Each shaver was used in a random order for the same patient. Tissue samples underwent testing for cellular proliferation, cellular concentration, number of colony-forming units (CFU), live/dead assay, fluorescence-activated cells sorting (FACS) analysis, cytokine analysis, and growth factor analysis. Shaver A produced more CFUs compared to Shaver B (210.3 vs. 125.9; *p* < 0.001). At 3 weeks, cells collected via Shaver A had greater cellular proliferation (0.35 vs. 0.51; *p* < 0.001) as well as more viable cells (214,773 vs. 132,356 cells/gram; *p* < 0.001). Tissue collected with Shaver B had greater amounts of the cytokines MMP-1 (3741 vs. 5500 pg/mL; *p* < 0.001), MMP-3 (1131 vs. 1871 pg/mL; *p* < 0.001), and MMP-13 (179 vs. 401 pg/mL; *p* < 0.001), while those collected with Shaver A had greater vascular endothelial growth factor (VEGF) (47.8 vs. 9.0 pg/mL; *p* < 0.05). Arthroscopic shavers are capable of harvesting and processing CTP cells from subacromial bursal tissue. Different shavers may produce different yields of viable CTP cells.

## 1. Introduction

Biologic augmentation of rotator cuff repairs provides a means to address the poor healing rates associated with this pathology [1,2,3,4,5,6]. Recent research has investigated the utility of different types of biologic augmentation such as platelet concentrates, connective tissue progenitor (CTP) cells, and growth factors [2,7,8,9,10]. In 2014, Heringou et al. showed that rotator cuff repairs augmented with bone-marrow-derived CTP cells showed greater healing potential and lower re-tear rates compared to those treated without augmentation [7].

While bone marrow is the most commonly used source of CTP cells, studies have shown the presence of CTP cells in the subacromial bursa [11,12,13,14]. Morikawa et al. showed that, compared to CTP cells derived from bone marrow aspirate, those derived from the subacromial bursa showed significantly increased differentiation ability and gene expression [2]. As our understanding of the utility of CTP cells in augmenting rotator cuff repairs grows, there is a need to optimize the methods by which these cells are harvested and then utilized. In 2020, Morikawa et al. demonstrated mechanical breakdown of bursal tissue with scissors resulted in similar amounts of nucleated cells compared to bursal tissue broken down with collagenase [1].

With the prevalence of the usage of arthroscopic shavers during rotator cuff repair, they present a potential tool for both the collection and preparation of CTP cells from bursal tissue. A recent study by Shin et al. demonstrated no difference in the amount of CTP cells derived from the anterior fat pad of the knee collected with a motorized shaver when compared to samples collected through rongeur biopsy [15]. However, Ferro et al. showed CTP cells collected with an arthroscopic shaver show increased proliferative capabilities when compared with those collected through direct biopsy [16]. As such, the arthroscopic shaver functions to both collect and process proliferative CTP cells, which can be used for biologic augmentation of rotator cuff repairs, without necessitating dedicated instrumentation for the preparation of these cells.

These shavers vary in blade design, torque, load capacity, and overall design [17]. To date, there have been no studies looking at the effectiveness of different shavers at collecting and processing bursal CTP cells for augmentation. The primary goal of this study is to determine if arthroscopic shavers are able to collect and process CTP cells. The secondary goal is to evaluate for any differences between the proliferative yields of the tested shavers. We hypothesize that both arthroscopic shavers will be able to effectively collect and process CTP cells from subacromial bursal tissues. We believe that both shavers will be capable of both collecting and processing CTP cells from subacromial bursal tissues with similar efficacy.

## 2. Materials and Methods

### 2.1. Patient Selection

This study had prior approval by the institutional review board before the initiation of the study. Nonconsecutive patients were enrolled from a single surgeon’s practice from March 2021 to June 2021. Patients were considered eligible for the study if they were over 18 years of age and were undergoing primary or revision arthroscopic rotator cuff repair. Vulnerable patient populations, such as prisoners or pregnant women, were excluded from the study. Exclusion criteria additionally excluded patients with current shoulder infection, active smokers, those with systemic inflammatory or rheumatologic disease, or history of radiation/chemotherapy due to potential effects on tissue.

### 2.2. Harvesting of Subacromial Bursa Tissue

**Figure 1:**
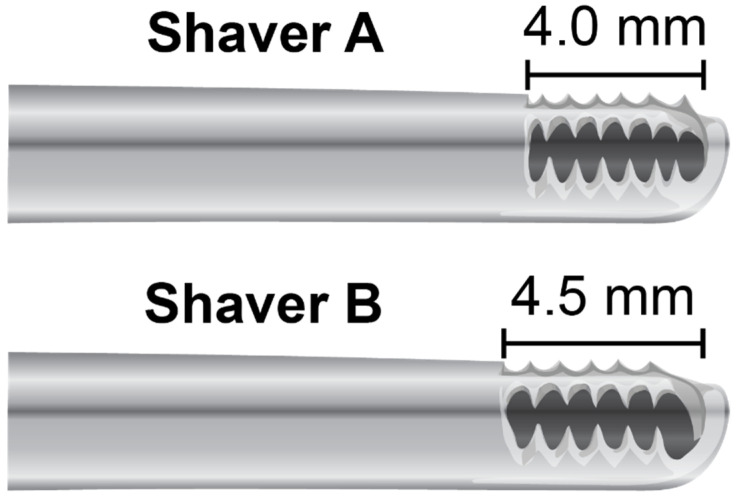
Shaver A (Arthrex Excalibur AR8400-EX a 6-toothed shaver with 4.0 mm tip and Shaver B (Smith & Nephew Incisor Plus Elite) with a 5-toothed 4.5 mm tip.

All procedures were performed with patient in a beach chair position. After arthroscopic evaluation of the subacromial bursa through the lateral viewing portal, partial subacromial debridement was performed via an anterior portal with two oscillating shavers (Figure 1; Shaver A = Arthrex Excalibur 4.0 mm AR-8400EX at 1750 rpm; Shaver B = Smith & Nephew Dyonics 4.5 mm Incisor Plus Elite Blade #7210976 in mode 2, speed 8). These shavers were chosen for similar size and rotary speed. Shaver A utilizes 6-toothed blade while Shaver B is 5-toothed. Information of further geometric specifications and blade torque are unavailable. Bursal tissue was collected from the same section of bursa for both shavers. Prior to study initiation, the order of arthroscopic shavers to be utilized for tissue collection in each patient was randomized. A collection device (GraftNet Autologous Tissue Collector; Arthrex, Naples, FL, USA) was attached to the suction on each shaver prior to debridement. When the collection device was full, the extracted bursa was measured in a 3 cc syringe until 1 cc of bursa was collected. After 1 cc of bursa was collected using the first shaver, a new collection device was attached to the second shaver and collection of 1 cc of bursa was repeated. Samples were placed in separate sterile specimen cups with saline and immediately transported to the laboratory for processing in a laminar flow hood. Samples were weighed upon their arrival, before 50 mg of tissue was placed into a well of a Corning Primaria Multiwell 24-well plate with 1 mL of complete Dulbecco’s Modified Eagle’s Medium (1×; Gibco, Life Technologies Limited, Paisley, UK), containing 10% fetal bovine serum (Gibco) and 1% penicillin/streptomycin. The dishes were cultured in a humidified incubator at 37 °C at 5% CO_2_. Culture medium was replaced twice a week thereafter. All laboratory measurements were performed by a single investigator.

### 2.3. Colony-Forming Units

A colony-forming unit (CFU) was defined as a cluster of 8 or more cells [18]. For all samples, cultures were checked on a daily basis for evidence of colony formation by a trained technician using an inverted-phase-contrast microscope (Nikon Eclipse TS 100, Nikon Corporation, Tokyo, Japan) at 10× magnification. Upon the appearance of colonies, the number of colonies was counted and recorded using the microscope. Each 100 mm culture dish was divided into four quadrants and the number of colonies in one of these quadrants was counted. This number was then multiplied for 4 to give the total number of colonies in each 100 mm culture dish.

### 2.4. Cellular Concentration

After 3 weeks of incubation, the number of cells that migrated out of the tissue and divided were counted. Three of the wells were trypsinized using sterile 0.5% trypsin/ethyl-enediaminetetraacetic acid (EDTA) to release the cells and then cells were resuspended in complete medium for counting. A total of 100 µL of cellular suspension was added to a cuvette containing 9.9 mL of 0.9% NaCl solution and counted using a Z1 Coulter Counter. Cellular concentration (cells/gram) was then calculated by dividing the number of cells by the total mass of plated tissue.

### 2.5. Cellular Proliferation

Cellular proliferation was evaluated after 3 weeks in culture. Proliferation was determined using the XTT (2,3-bis(2-methoxy-4-nitro-5-sulfophenyl)-5-[(phenylamino) carbonyl]-2H-tetrazolium hydroxide) assay (Roche Diagnostics, Mannheim, Germany). Cultures were incubated for 24 h in XTT labeling mixture before the absorbance at 450 nm with a reference wavelength of 650 nm was measure with a plate read (BioTek, Bad Friedrichshall, Germany) [2,19]. This assay is based on the mitochondrial conversion of tetrazolium salt XTT to a soluble formazan salt in metabolically intact and active cells.

### 2.6. FACS Analysis

Upon reaching near confluence (approximately 3 weeks in culture), fluorescence-activated cell sorting (FACS) analysis was completed on the cells that grew out of the tissue. Cells were assessed for the presence of surface markers CD90, CD105, and CD73 and the absence of CD45 and CD31 based on the consensus markers set by the International Society for Cellular Therapy for mesenchymal stem cells [20]. Cells were trypsinized using sterile 0.5% trypsin/EDTA. The Z1 Coulter Counter was used to determine cellular concentrations and 1 million cells were resuspended in 100 µL of staining buffer (1× phosphate-buffered saline with 1% FBS and 1% human serum) containing a fluorescein isothiocyanate or phycoerythrin antibody. Antibodies were obtained from BS Biosciences (San Diego, CA, USA). Surface markers were measured with a BD LSR II flow cytometer and data were analyzed using BD FACS-Diva software (BD Biosciences).

### 2.7. Live/Dead Assay

The viability of the tissue collected by each shaver was assed using a live/dead assay performed at time 0 and after 3 weeks in culture. The tissue was incubated in 5 mM calcein and 10 mg/mL propidium iodide (Thermo Fisher Scientific, Waltham, MA, USA) in 1× phosphate-buffered saline to stain for live and dead cells within the tissue. After 30 min, the tissue was washed two times with phosphate-buffered saline and the green or red fluorescence was visualized and quantified using a Leice DMI 6000 B fluorescent microscope (Leice Microsystems, Buffalo Frove, IL, USA). Images were randomly taken from three sections of the culture well and the number of live and dead cells were counted.

### 2.8. Cytokine and Growth Factor Analysis

Cytokines and growth factors were quantified in media at the initiation of the culture (time 0) and after 96 h in culture [21]. Time 0 media was collected right after tissue was placed in media. Excreted inflammatory cytokines chosen for degenerative tendon analysis included interleukins (IL): IL-1β, IL-1rα, IL-6, and tumor necrosis factor alpha (TNF-α) [22]. Collagenases, enzymes of the metalloproteinase (MMP) family can cleave intact fibrillar collagen and play an important role in connective tissue turnover. Therefore, an increase in net MMP activity is likely to indicate matrix degradation, which may represent part of the remodeling process in wound healing. For this reason, MMP-1, MMP-3 and MMP13 were chosen for analysis [23]. Vascular endothelial growth factor (VEGF) was chosen as it a marker for angiogenesis and cellular proliferation in human tendon [24,25]. Active concentrations were determined using enzyme-linked immunosorbent assays (ELISA).

### 2.9. Statistics

Descriptive statistics included mean and standard deviation to characterize the shaver groups. Differences between the shavers were examined with mixed-effects linear models. A random intercept was used to account for the correlation introduced with patient specific tissue samples. Marginal mean values for each comparison of interest were reported as mean difference with corresponding 95% confidence intervals. Data was then substratified by gender and primary versus revision surgery. All analyses were performed using Stata 15.1 software (StataCorp. 2017. Stata Statistical Software: Release 15. StataCorp LLC, College Station, TX, USA).

## 3. Results

Samples were collected from 10 patients (Table 1). There were six male patients and four female patients. The average age of the patients was 54.1 years old (range 39 to 67 years old). Of the patients included, eight underwent primary rotator cuff repair and two underwent revision rotator cuff repair.

### 3.1. Colony Forming Units

**Figure 2:**
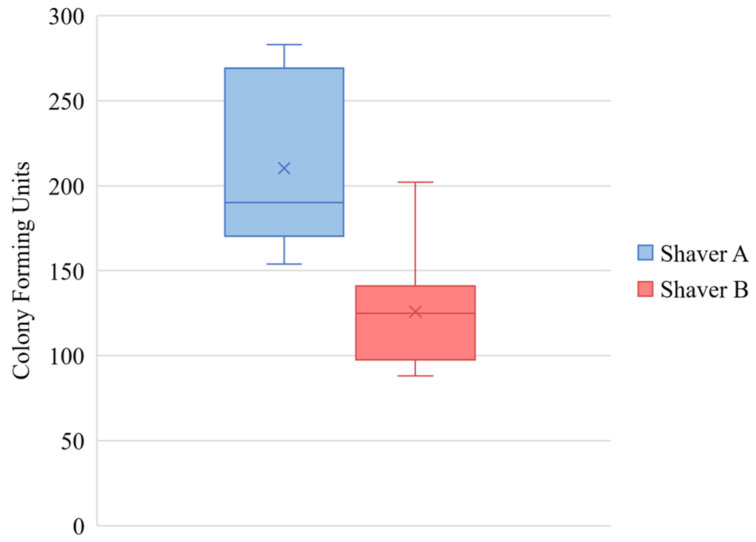
Number of colony-forming units after 14 days in culture demonstrating significantly more CFUs formed by the tissue collected by Shaver A compared to Shaver B (*p* < 0.001). The values are represented as the mean (“X” marker), median (line), interquartile range (box), and range (whiskers).

Figure 2 demonstrates that more colonies were formed after 14 days by tissue collected by Shaver A versus Shaver B, 210.3 ± 50 versus 129 ± 34 colonies, respectively (*p* < 0.001). There was no effect of gender (*p* = 0.852) or revision surgery status (*p* = 0.15) on number of colony-forming units.

### 3.2. Cellular Concentration

**Figure 3:**
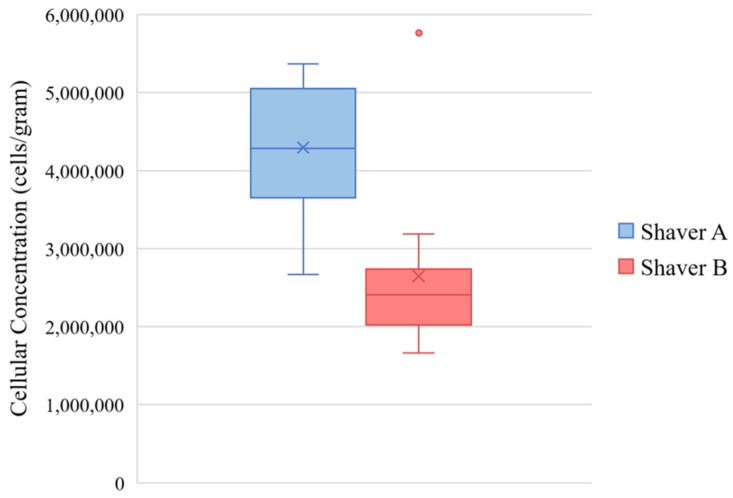
Cellular concentration of bursal cells. (*p* < 0.001). The values are represented as the mean (“X” marker), median (line), interquartile range (box), and range (whiskers). One outlier is expressed as a dot outside of the range of the Shaver B group.

Following 3 weeks in culture, bursa samples obtained from Shaver A (4.30 × 10^6^ ± 1.18 × 10^6^ cells/gram) demonstrated a significantly higher concentration of cellular outgrowth compared to Shaver B (2.65 × 10^6^ ± 8.50 × 10^5^ cells/gram) (*p* < 0.001). Figure 3 shows the cellular concentrations. There was no effect of revision surgery status cellular concentration (*p* = 0.055). Tissue from female patients had a higher concentration of cells compared to males (*p* = 0.007). Additionally, tissue collect from female patients with Shaver B had a greater cellular concentration compared with samples collected with Shaver A (*p <* 0.001).

### 3.3. Cellular Proliferation

**Figure 4:**
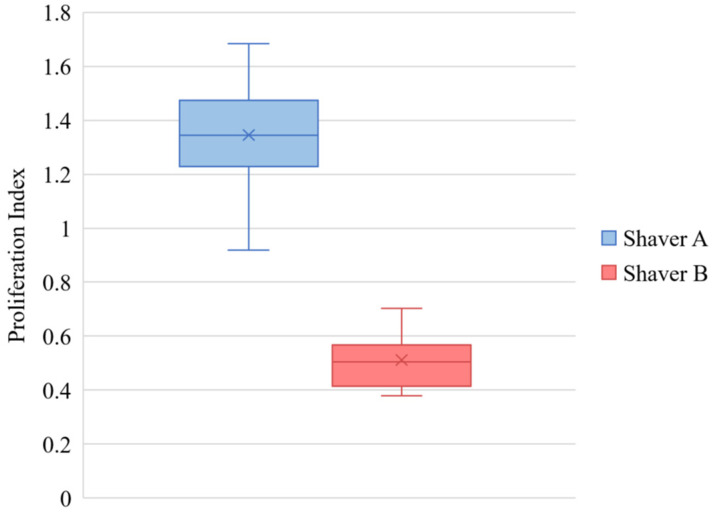
Cellular proliferation of bursal cells demonstrating a higher mean proliferation index of from cells isolated from Shaver A compared to Shaver B (*p* < 0.001). The values are represented as the mean (“X” marker), median (line), interquartile range (box), and range (whiskers).

Following 3 weeks in culture, bursa samples obtained from Shaver A (1.36 ± 0.21 demonstrated a significantly higher proliferation index compared to Shaver B (0.51 ± 0.10) (*p* < 0.001). Figure 4 shows the cellular proliferation. There was no effect of gender (*p* = 0.064) or revision surgery status (*p* = 0.986) on proliferation.

### 3.4. FACS Analysis

**Figure 5:**
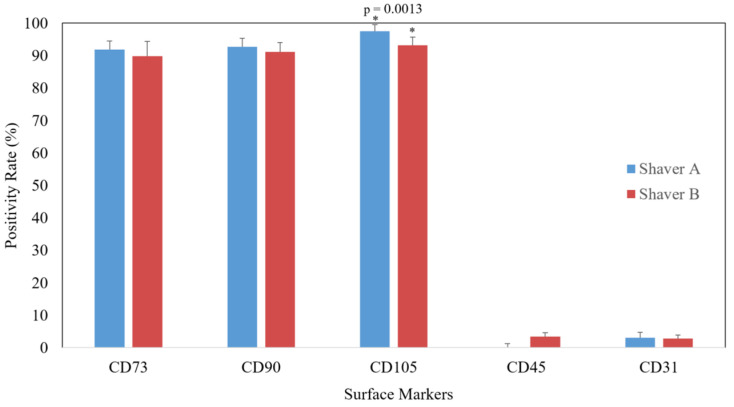
Positivity rate for CTP-specific surface markers. Both shavers demonstrated high positivity rates of CTP-specific markers (CD73, CD90, and CD105) and low positivity rates of negative markers (CD45 and CD31). Values presented as mean positivity rate with standard deviation bars. * = *p* < 0.05.

Subacromial bursal cells isolated from the tissue collected by both shavers expressed high positivity for CTP cells (CD73, CD105, and CD90) and low positivity for hematopoietic surface markers (CD45 and CD31). This suggests that both shavers were able to appropriately isolate CTP cells [20]. Shaver A harvested cells did have significantly more (97.6%) CD105 expression compared Shaver B (93.4%) (*p* = 0.0013). There was no significant difference in any of the other surface markers (Figure 5). There was no effect of gender (*p* = 0.946; *p* = 0.506; *p* = 0.123) or revision surgery status (*p* = 0.603; *p* = 0.488; *p* = 0.362) on surface marker expression.

### 3.5. Live/Dead Assay

**Figure 6:**
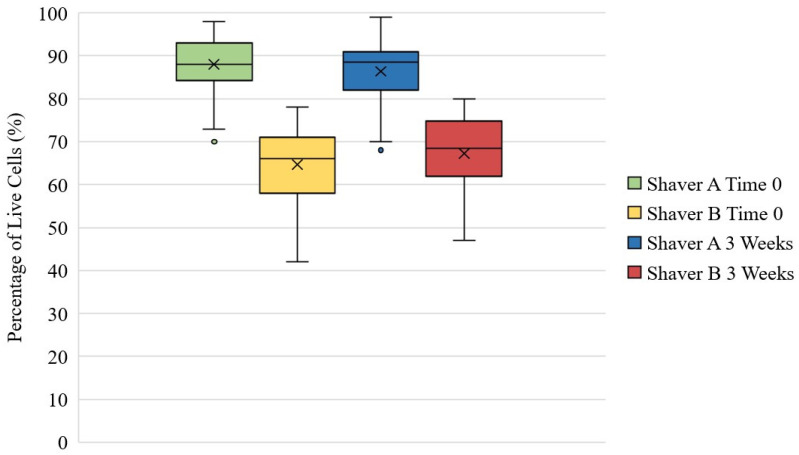
Percentage of live cells in tissue collect by Shaver A and Shaver B at Time 0 and after 3 weeks in culture. At each time point, the number of live cells in the tissue collected by Shaver A was greater than Shaver B at both Time 0 and 3 weeks (*p* < 0.001).The values are represented as the mean (“X” marker), median (line), interquartile range (box), and range (whiskers). Outliers expressed as dots outside of range.

**Figure 7:**
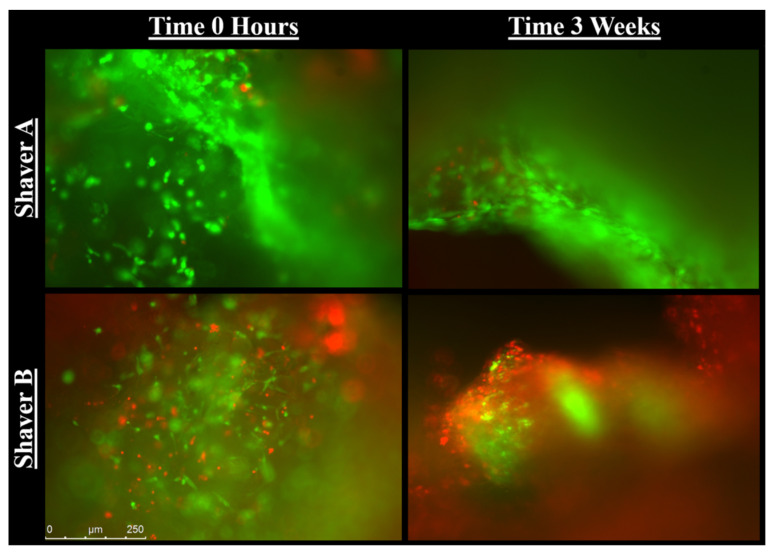
Representative images of live/dead assay. Living cells are labeled with green immunofluroescence while dead cells are labelled with red. (Magnification, 10×).

Figure 6 represents that at both time zero and three weeks, there were significantly more live cells in the tissue collected from Shaver A compared to Shaver B (*p* < 0.001). At time zero, Shaver A collected 87.99 ± 6.12% live cells compared to 64.70 ± 8.33% live cells collect from Shaver B. There was no effect of gender (*p* = 0.12) or revision surgery status (*p* = 0.965) on ratio of live to dead cells at time zero. At 3 weeks, Shaver A collected 86.35 ± 6.89% live cells compared to 67.29 ± 8.72% live cells collect from Shaver B. Figure 7 is representative images of Live/Dead assay. While there was no difference in live cell ratio based on revision status at 3 weeks (*p* = 0.737), there was a difference based on gender (*p* = 0.005) with females having a greater concentration of live cells when compared to males. In females, Shaver B was found to collect a greater ratio of live to dead cells (*p* < 0.001) at 3 weeks when compared to Shaver A.

### 3.6. Cytokine and Growth Factor Analysis

Table 2 shows the mean concentrations (pg/mL) and the standard deviation of selected cytokines and growth factors at 0 h and 96 h in the culture media.

At time zero, IL-1Ra, MMP-1 and VEGF were higher and MMP-3 was lower in concentration in media surrounding tissue collected by Shaver A (*p* < 0.05). There was no significant difference in IL-1β and IL-6 between the shavers (*p* > 0.05). The differences in TNF-α and MMP-13 concentrations could not be determined.

At 96 h in culture, significantly more VEGF and significantly less IL-1β, IL-6, MMP-1, MMP-3, MMP-13 were released into the media of tissue collected by Shaver A compared to Shaver B (*p* < 0.05) There was no significant difference in IL-1Ra released into media between the two shavers (*p* > 0.05) The difference in in TNF-α concentrations could not be determined.

Revision bursal tissue had higher levels if IL-6 expression at 96 h (*p* = 0.043) but not at 0 h (*p* = 0.158) when compared with primary tissue. There were no differences based on gender or revision status for the remaining cytokines or growth factors at either timepoint.

### 3.7. Post Hoc Power Analysis

A post hoc power analysis was performed for the primary outcome measure which was CFUs. With an α of 0.05, this outcome was found to have a β = 0.992.

## 4. Discussion

Our study found that both arthroscopic shavers were able to collect and process subacromial bursal tissue for the augmentation of rotator cuff repairs. Both shavers provided excellent yields of proliferative CTP cells from subacromial bursal tissues. Cells from each of these shavers showed a large number of mesenchymal stem-cell surface markers (CD90, CD105, and CD73) and low levels of hematopoetic-cell surface markers (CD45 and CD31) indicating that both shavers were capable of isolating CTP cells from bursal tissue. While both fulfilled this function effectively, there were some differences in the final cellular yield. Shaver A produced a greater number of CFUs in compared to Shaver B. Additionally, the cells from Shaver A showed greater cellular outgrowth, proliferation, and viability. Tissue collected with Shaver B had greater amounts of the cytokines MMP-1, MMP-3, and MMP-13, while those collected with Shaver A had greater levels of VEGF.

While these results did confirm that both arthroscopic shavers were able to collect and process CTP cells from the subacromial bursa, it did also find differences in the resultant cells. Previous studies have found that different blade designs and oscillating speeds have a great impact on the tissue resection capabilities of a shaver [17,26]. It is unclear which aspects of each shavers’ design and functionality create the discrepancies in CTP yield. Additionally, while similar shaver speeds and sizes were used for this study, the manufacturers do not report thorough design specifications of each shaver, such as torque used or detailed head geometry. While Shaver A utilizes a 6-toothed blade and Shaver B uses a 5-toothed blade, there is no current literature comparing the effects of number of teeth on ability to isolate viable tissue. Determining the cause of these differences in outcome is important for device manufacturers to help develop future shavers that may be even more effective at processing bursal tissue.

Our study did demonstrate that there may be a difference in quality of tissue harvested and processed using arthroscopic shavers based on both gender and revision surgery status. Most interestingly, our data showed that females had a greater cellular concentration compared to men overall, and that cellular concentration was greater in females with Shaver B rather than Shaver A. This is opposite of the effect seen in the overall subject pool. Further investigation is warranted to understand what effect gender has on the composition of subacromial bursal tissue. Furthermore, our data found that tissue collected from revision cases had a lower cellular concentration when compared with primary cases. Additionally, revision cases had a greater concentration of IL-6 compared with primary cases. As our study only contained two revision cases, further investigation is needed to determine the composition of revision bursal tissue when compared with primary bursal tissue.

CTP cells have proven to be a powerful source of biological augmentation for rotator cuff repairs [7]. The subacromial bursa provides an easily accessed source of CTP cells when performing an arthroscopic rotator cuff repair [12,13,14]. It is important to optimize the methods for collecting CTP cells from subacromial bursal tissue in a way that is practical for the treating surgeon. Previous studies have shown that different methods for processing subacromial bursal tissue have significant effects of yield of viable CTP cells (Morikawa, 2020). Shavers are commonly used tools in arthroscopic rotator cuff repair and, as shown in this study, are capable of collecting and processing CTP cells from subacromial bursal tissue. Tissue collected from arthroscopic shavers can then be used for the biological augmentation of rotator cuff repairs within the same patient.

This study was not without limitations. Firstly, this study only compared two types of arthroscopic shavers. Given the significant differences between the two, it can be inferred that other brands of shavers may be better or worse at producing viable cells. While the differences observed between shavers may be attributed to device characteristics such as blade shape, torque, and geometry, information on these characteristics for the shavers used in this study are not made available and could not be obtained from either company. Further testing with other shavers would need to be carried out to elucidate these differences. Furthermore, this was not a blinded study, and the brand of shaver being used was known to the treating surgeon. We combated this by using both shavers in each patient and randomizing the order with which each shaver was used. A further limitation of this study is the limited sample size which reduces both the generalizability of this data and the ability to determine the effects of patient factors such as age on the results. Additionally, other demographic data such as medical comorbidities, race, hand-dominance were not evaluated for the patients included in this study. Additionally, the in vitro proliferation of the samples may not reflect the cellular activity that occurs in vivo. To date, the behavior of subacromial bursa-derived cells in vivo is not known. Finally, while there were differences in the ultimate yield of each shaver, it is unknown the minimum amount of CTP cells needed to result in a beneficial augmentation of a repair, and as such if more CTP cells is necessarily clinically better.

## 5. Conclusions

This study demonstrated that arthroscopic shavers are capable at collecting and processing CTP cells from subacromial bursal tissue in human subjects. Surgeons augmenting rotator cuff repairs with CTP cells derived from bursal tissue can utilize arthroscopic shavers for both the collection and preparation of this tissue.

## Figures and Tables

**Table 1:** Patient demographic information.

Patient Number	Age	Gender	Surgery
1	46	Male	Rotator Cuff Repair
2	39	Male	Rotator Cuff Repair
3	54	Male	Revision Rotator Cuff Repair
4	57	Female	Rotator Cuff Repair
5	55	Female	Rotator Cuff Repair
6	51	Male	Rotator Cuff Repair
7	49	Female	Rotator Cuff Repair
8	61	Male	Revision Rotator Cuff Repair
9	67	Female	Rotator Cuff Repair
10	62	Male	Rotator Cuff Repair

**Table 2:** Concentrations (pg/mL) of cytokines and growth factors at time 0 h and 96 h.

	Shaver A Time 0	Shaver B Time 0	*p*-Value	Shaver A Time 96 h	Shaver B Time 96 h	*p*-Value
IL-1β	0.20 ± 0.28	0.23 ± 0.36	0.610	0.14 ± 0.16	0.27 ± 0.19	0.001
IL-1Ra	57.31 ± 165.99	31.16 ± 68.15	0.048	152.43 ± 151.82	147.40 ± 113.84	0.785
IL-6	2.02 ± 5.44	3.46 ± 7.42	0.256	20.61 ± 32.88	70.05 ± 122.88	<0.001
TNF-α	ND	0.09 ± 0.39	N/A	ND	0.13 ± 0.49	N/A
MMP-1	44.85 ± 63.49	26.22 ± 53.48	0.037	3741.00 ± 2842.63	5500.11 ± 2867.97	<0.001
MMP-3	527.21 ± 318.48	765.32 ± 473.88	0.001	1130.87 ± 408.29	1871.05 ± 1268.86	<0.001
MMP-13	ND	18.89 ± 31.77	N/A	178.85 ± 89.70	401.23 ± 169.52	<0.001
VEGF	47.84 ± 129.54	8.96 ± 11.43	0.009	345.54 ± 182.43	169.66 ± 160.51	<0.001

Data are expressed as mean ± standard deviation. VEGF = vascular endothelial growth factor; ND = nondetectable; N/A = Not Applicable.
